# Effect of Sucrose Concentration on *Rhaponticum carthamoides* (Willd.) Iljin Transformed Root Biomass, Caffeoylquinic Acid Derivative, and Flavonoid Production

**DOI:** 10.3390/ijms232213848

**Published:** 2022-11-10

**Authors:** Ewa Skała, Monika Anna Olszewska, Joanna Makowczyńska, Agnieszka Kicel

**Affiliations:** 1 Department of Biology and Pharmaceutical Botany, Medical University of Lodz, Muszynskiego 1, 90-151 Lodz, Poland; 2 Department of Pharmacognosy, Medical University of Lodz, Muszynskiego 1, 90-151 Lodz, Poland

**Keywords:** growth cycle, chlorogenic acid, 3,5-di-*O*-caffeoylquinic acid, 4,5-di-*O*-caffeoylquinic acid, 1,4,5-*O*-tricaffeoylquinic acid

## Abstract

*Rhaponticum carthamoides* (Willd.) Iljin is a rare, pharmacopoeial, and medicinal plant, endemic to Siberia and endangered due to the massive collection of raw material from the natural habitat. The aim of the current study was to estimate the effect of sucrose concentration (0–7%) on *R. carthamoides* transformed root growth and on caffeoylquinic acid derivative (CQA) and flavonoid production. Sucrose in higher concentrations may induce osmotic stress and thus may affect secondary metabolism in plants. It was revealed that sucrose concentration influenced *R. carthamoides* transformed root biomass and modified the phenolic compound metabolic pathway. However, the dynamics of both processes varied significantly. The optimal sucrose level was different for biomass accumulation and the biosynthesis of specialized metabolite. The highest dry weight of roots was achieved for 7% sucrose (31.17 g L^−1^ of dry weight), while 1% sucrose was found to be optimal for phenolic acid and flavonoid production. Considering the dry weight increase and metabolite accumulation, 3% sucrose was revealed to give optimal yields of CQAs (511.1 mg L^−1^) and flavonoids (38.9 mg L^−1^). Chlorogenic acid, 3,5-, 4,5-di-*O*-caffeoylquinic acids, 1,4,5-*O*-tricaffeoylquinic acid, and a tentatively-identified tricaffeoylquinic acid derivative 1 were found to be the most abundant specialized metabolites among the identified CQAs. Our findings indicate that *R. carthamoides* transformed roots may be an efficient source of CQA derivatives, with valuable health-promoting activities.

## 1. Introduction

Plants have a rich history as diet supplements and pharmaceuticals, thanks to the wide range of their specialized metabolites demonstrating health-promoting properties. They have been used for thousand years in traditional medicine, due to their multidirectional biological activity. A considerable increase in their application as traditional medicines has been observed during the last few decades. Additionally, pure compounds isolated from plant extracts offer promise as treatments, due to them being less toxic and more economical than synthetic compounds [1].

One of the more numerous groups of plant specialized metabolites known to demonstrate biological properties are polyphenolic compounds with phenolic acids [2]. Among these are the caffeoylquinic acid derivatives (CQAs), promising phytotherapeutic agents in the daily human diet [3]. They offer many health benefits, including antioxidant, anti-inflammatory, antimicrobial, neuro- and hepatoprotective, antihypertensive, antihyperglycemic, and anticancer activities [4,5]. CQAs are present in a number of plant species, such as those from the Asteraceae family [5]. One such species is *Rhaponticum carthamoides* (Willd.) Iljin [6]. This is an endemic plant that occurs in alpine and subalpine meadows and tundra in West and East Siberia, Altai and Sajan Mountains, Mongolia, and Kazakhstan, and which is cultivated in Eastern and Central Europe [6,7,8]. *R. carthamoides* is classified as a rare, endangered species, due to the massive collection from its natural habitat [9]. The herbal materials are the roots and rhizome, *Rhapontici carthamoidis rhizomata cum radicibus*. *R. carthamoides* is monographed in the official pharmacopoeias of some Eastern European countries as a tonic and adaptogen agent [7].

Due to the healing properties of plants and their specialized metabolites, researchers have long been looking for alternative methods for harvesting plants from the natural environment or plantations. This is especially important when the raw materials are roots or/and rhizomes, as their acquisition requires the destruction of the mother plant. Additionally, the secondary metabolism of plants, and thus the content of bioactive compounds, are influenced by numerous environmental factors (drought, temperature, light, and salinity) and the developmental stage of plants [10]. Green biotechnology, which uses genetic engineering to obtain in vitro transgenic plant cultures, may be a profitable approach for the production of raw materials and pharmacologically-active compounds under sterile, strictly-defined conditions, regardless of the season or climate. The transformed roots obtained by *Rhizobium rhizogenes* transformation may be an efficient and cost-effective source of various specialized metabolites [11]. To date, numerous protocols have been developed for obtaining and optimizing the growth of transformed roots of medicinally important plants, coming from different climatic zones and often threatened with extinction, as well as for the production of the various biologically active compounds or recombinant protein made by them [11,12]. Transformed roots offer genetic stability, fast biomass growth in media without growth regulators, and a relatively potent production of the specialized metabolites [11]. Additionally, the transformed roots may also be used as a tool to regenerate whole transgenic pRi-transformed plants, where the root system is strongly developed and the roots show long and numerous lateral branches [13]. pRi-transformed plants often offer a higher production of specialized metabolites than non-transgenic plants.

Many studies have indicated that plant in vitro culture can be a profitable approach for CQA production [4,14]. Our earlier studies found *R. carthamoides* transformed roots and pRi-transformed plants to be a rich source of CQAs, especially chlorogenic acid, and di- and tricaffeoylquinic acids [15,16]; the latter being quite rare natural compounds. However, to obtain cultures that produce high levels of these valuable specialized metabolites, the culture conditions need to be optimized with regard to both biomass and specialized metabolite accumulation. The specialized metabolite production can be enhanced by plant in vitro culture, by choosing the type of medium, growth conditions, elicitor treatments, and by over-expressing the genes encoding the enzymes involved in the specialized metabolite biosynthesis pathway, or by introducing new genes or silencing others [4,17,18].

Carbohydrates can affect both plant biomass accumulation and specialized metabolite production by plant in vitro culture. Sucrose may induce osmotic or carbonyl stress as an elicitor, especially when it is used at high concentration [19]; this may affect the secondary metabolism and thus increase specialized metabolite production in the transformed roots of many plant species, such as the production of pyranocoumarins in *Angelica gigas* [20], ginsenosides in *Panax quinquefolium* [21], baicalein in *Scutellaria baicalensis* [22], and iridoid glycosydes in *Picrorhiza kurroa* [23]. The results of Solfanelli et al. [24] showed that carbohydrates influence various genes encoding enzymes involved in specialized metabolite biosynthetic pathways, e.g., flavonoid and anthocyanin biosynthesis in *Arabidopsis thaliana*.

Therefore, the aim of the present study was to determine, for the first time, the influence of sucrose concentration (0–7%) on the growth and production of certain CQAs and flavonoid monoglycosides in transformed roots of *R. carthamoides*. The changes in biomass and selected specialized metabolite accumulation during the growth cycle of the transformed roots were also evaluated.

## 2. Results

### 2.1. Influence of Sucrose Concentration on Transformed Root Growth

**Figure 1:**
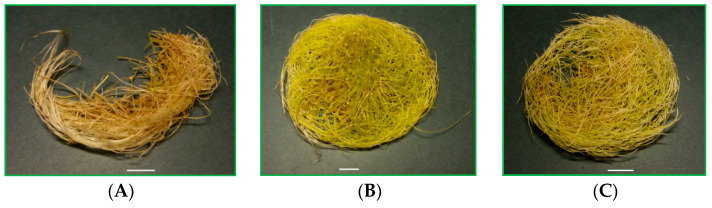
*R. carthamoides* transformed roots cultured in 50 mL of liquid WPM medium in a 300 mL Erlenmeyer flask in light conditions (16/8-h light/dark photoperiod) on a rotary shaker at 80 rpm (**A**) without sucrose, supplemented with (**B**) 3% sucrose, and (**C**) 7% of sucrose. *Bar* = 1 cm.

**Figure 2:**
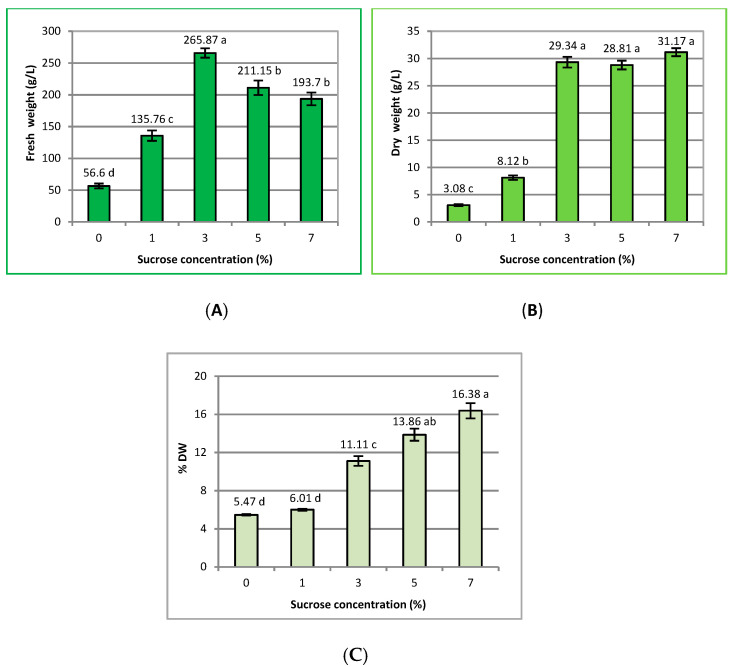
Effect of sucrose concentration (0–7%) on (**A**) fresh weight (g L^−1^), (**B**) dry weight (g L^−1^), and (**C**) % of dry weight of *R. carthamoides* transformed roots cultured in 50 mL of liquid WPM medium in a 300 mL Erlenmeyer flask, in light conditions (16/8-h light/dark photoperiod) on a rotary shaker at 80 rpm. The results are means values ± SE. The experiments were repeated three times (*n* = 9). Different letters signify significant differences between the sucrose concentrations (*p* < 0.05).

In the present study was tested the influence of sucrose concentration (0–7%) on the biomass accumulation of *R. carthamoides* transformed roots after five weeks of culture. It was observed that the transformed roots required sucrose for growth. The lowest biomass (56.6 g L^−1^ FW and 3.08 g L^−1^ DW) was found when the roots were grown in the medium without sucrose supplementation (Figure 1A and Figure 2A,B). The highest fresh weight of the transformed roots (265.87 g L^−1^) was observed in the presence of 3% sucrose (Figure 2A); this was an almost 14-fold increase of biomass compared to the initial weight. It was found that increases in sucrose concentration to 5% and 7% inhibited the growth of the roots and FW yielded 211.15 g L^−1^ and 193.7 g L^−1^, respectively (Figure 2A).

The dry weight of *R. carthamoides* transformed roots increased with the sucrose concentration up to 7% and achieved a maximal value of 31.17 g L^−1^ (Figure 2B). The growth index (initial weight to final biomass) also peaked at 7% sucrose, increasing 19-fold. The dry weight was slightly lower at sucrose concentrations of 3% and 5% (29.34 g L^−1^ and 28.81 g L^−1^, respectively) (Figure 2B).

### 2.2. Influence of Sucrose Concentration on CQA and Flavonoid Production

**Figure 3:**
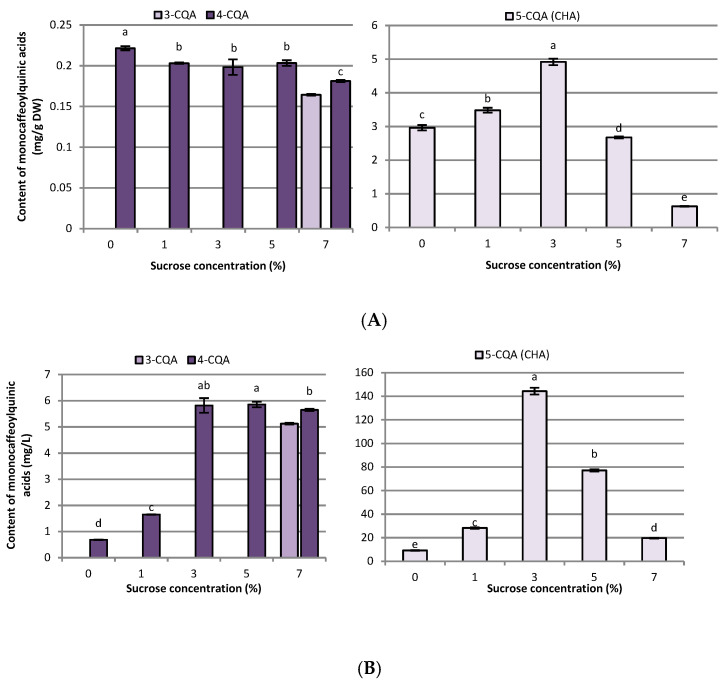
Effect of sucrose concentration (0–7%) on monocaffeoylquinic acid derivative contents in (**A**) mg g^−1^ DW and (**B**) mg L^−1^ in *R. carthamoides* transformed roots cultured in 50 mL of liquid WPM medium in a 300 mL Erlenmeyer flask, in light conditions (16/8-h light/dark photoperiod) on a rotary shaker at 80 rpm. The results are means values ± SE (*n* = 9). Different letters for the same metabolite signify significant differences between the sucrose concentrations (*p* < 0.05). 3-CQA-3-*O*-caffeoylquinic acid; 4-CQA-4-*O*-caffeoylquinic acid; 5-CQA-5-*O*-caffeoylquinic acid (CHA, chlorogenic acid).

**Figure 4:**
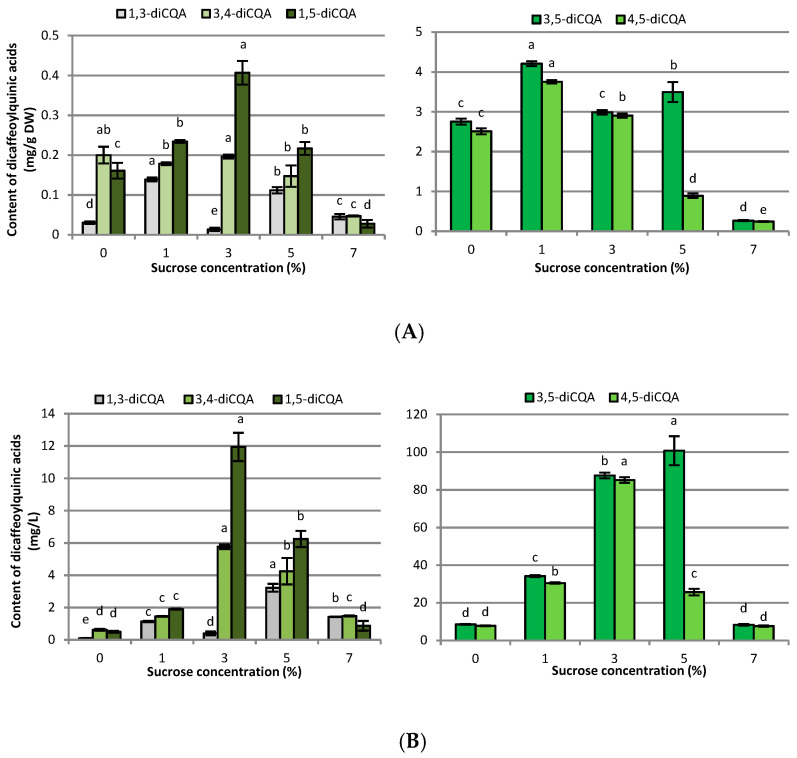
Effect of sucrose concentration (0–7%) on dicaffeoylquinic acid derivative contents in (**A**) mg g^−1^ DW and (**B**) mg L^−1^ in *R. carthamoides* transformed roots cultured in 50 mL of liquid WPM medium in a 300 mL Erlenmeyer flask, in light conditions (16/8-h light/dark photoperiod) on a rotary shaker at 80 rpm. The results are means values ± SE (*n* = 9). Different letters for the same metabolite signify significant differences between the sucrose concentrations (*p* < 0.05). 3,5-diCQA-3,5-di-*O*-caffeoylquinic acid; 4,5-diCQA-4,5-di-*O*-caffeoylquinic acid; 1,3-diCQA-1,3-di-*O*-caffeoylquinic; 3,4-diCQA-3,4-di-*O*-caffeoylquinic; 1,5-diCQA-1,5-di-*O*-caffeoylquinic.

**Figure 5:**
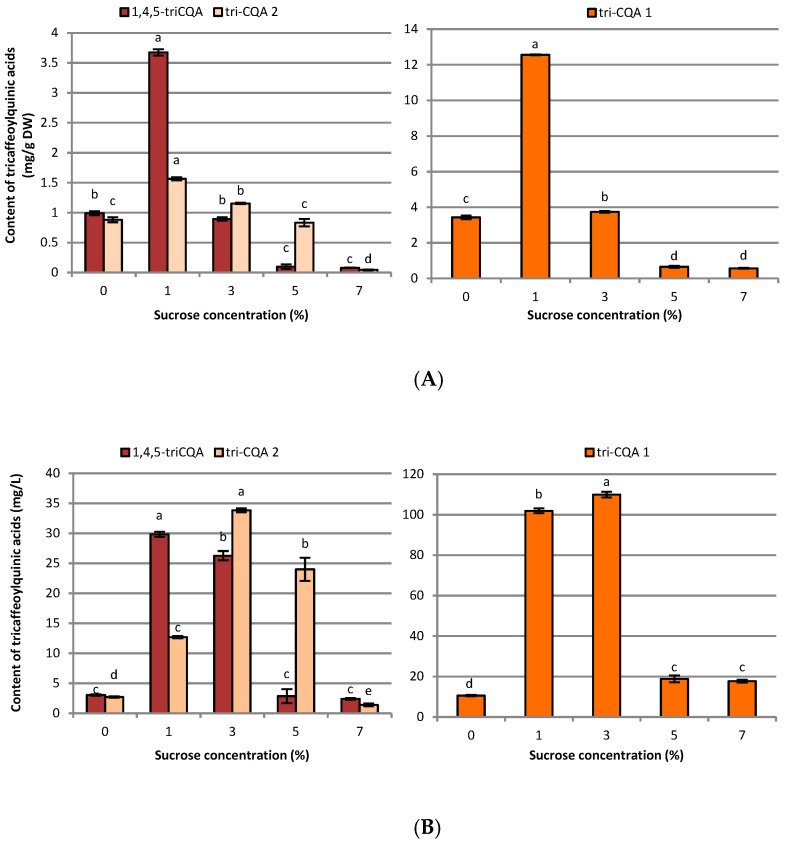
Effect of sucrose concentration (0–7%) on tricaffeoylquinic acid derivative contents in (**A**) mg g^−1^ DW and (**B**) mg L^−1^ in *R. carthamoides* transformed roots cultured in 50 mL of liquid WPM medium in a 300 mL Erlenmeyer flask, in light conditions (16/8-h light/dark photoperiod) on a rotary shaker at 80 rpm. The results are means values ± SE (*n* = 9). Different letters for the same metabolite signify significant differences between the sucrose concentrations (*p* < 0.05). 1,4,5-triCQA-1,4,5-tri-*O*-caffeoylquinic acid; tri-CQA 1 and tri-CQA 2-tricaffeoylquinic acid derivatives.

**Figure 6:**
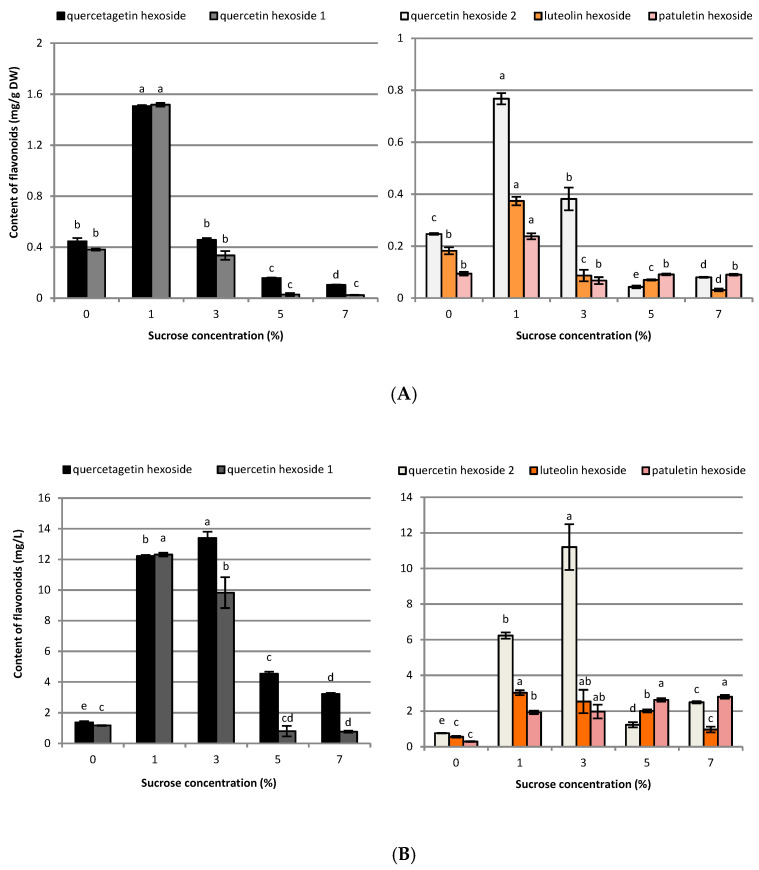
Effect of sucrose concentration (0–7%) on flavonoid contents in (**A**) mg g^−1^ DW and (**B**) mg L^−1^ in *R. carthamoides* transformed roots cultured in 50 mL of liquid WPM medium in a 300 mL Erlenmeyer flask, in light conditions (16/8-h light/dark photoperiod) on a rotary shaker at 80 rpm. The results are means values ± SE (*n* = 9). Different letters for the same metabolite signify significant differences between the sucrose concentrations (*p* < 0.05).

**Figure 7:**
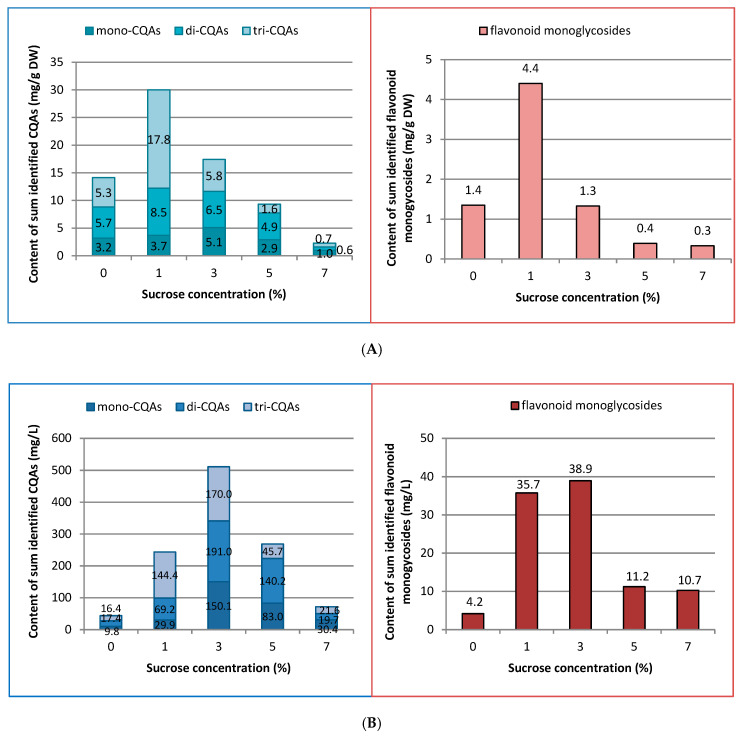
Effect of sucrose concentration (0–7%) on the total content of caffeoylquinic acid derivatives and flavonoid monoglycosides in (**A**) mg g^−1^ DW and (**B**) mg L^−1^ in *R. carthamoides* transformed roots cultured in 50 mL of liquid WPM medium in a 300 mL Erlenmeyer flask, in light conditions (16/8-h light/dark photoperiod) on a rotary shaker at 80 rpm.

**Figure 8:**
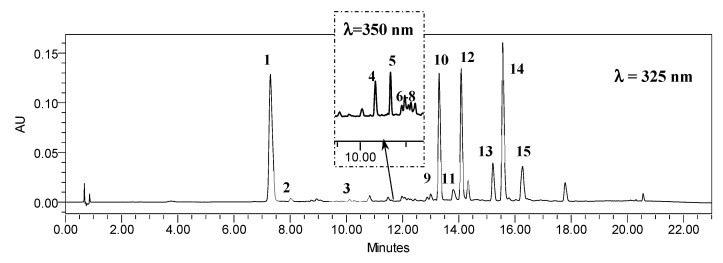
Representative HPLC-UV chromatogram of *R. carthamoides* transformed root extract recorded at 325 nm and 350 nm; **1**: 5-*O*-caffeoylquinic acid (5-CQA, CHA, chlorogenic acid); **2**: 4-*O*-caffeoylquinic acid (4-CQA); **3:** 1,3-di-*O*-caffeoylquinic acid (1,3-diCQA); **4**: quarcetagetin hexoside; **5, 6**: quercetin hexosides; **7**: luteolin hexoside; **8**: patuletin hexoside; **9**: 3,4-di-*O*-caffeoylquinic acid (3,4-diCQA); **10**: 3,5-di-*O*-caffeoylquinic acid (3,5-diCQA); **11**: 1,5-di-*O*-caffeoylquinic acid (1,5-diCQA); **12**: 4,5-di-*O*-caffeoylquinic acid (4,5-diCQA); **13**: 1,4,5-*O*-tricaffeoylquinic acid (1,4,5-triCQA); **14, 15**: tricaffeoylquinic acid derivatives.

**Figure 9:**
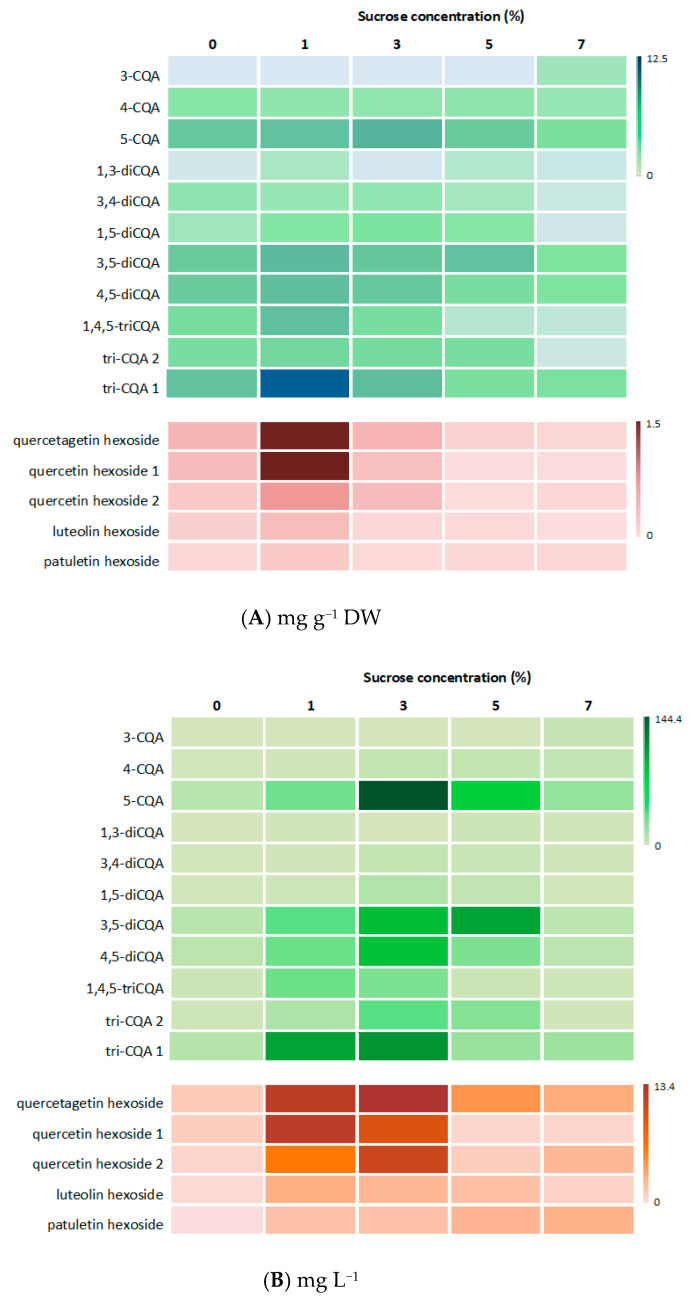
Heat map visualization of CQA and flavonoid content in (**A**) mg g^−1^ DW and (**B**) mg L^−1^ in *R. carthamoides* transformed roots cultured in liquid WPM medium supplemented with different sucrose concentrations (0–7%). Lighter colored boxes indicate a lower metabolite content. Darker colored boxes indicate a higher metabolite level.

HPLC-PDA analysis demonstrated that sucrose concentration (0–7%) significantly affected the level of eleven caffeoylquinic acid derivatives and five flavonoid monoglycosides in the transformed roots of *R. carthamoides* (Figure 3, Figure 4, Figure 5, Figure 6, Figure 7, Figure 8 and Figure 9). The total CQA content ranged from 2.30 mg g^−1^ DW to 30 mg g^−1^ DW (Figure 7A). The highest level of these compounds was observed for the roots cultured in the presence of 1% sucrose. The tricaffeoylquinic acid derivatives (tri-CQAs) were the predominant components (Figure 7A). The content of the main tentatively-identified tri-CQA 1 derivative (t_R_ = 15.6 min) was 12.55 mg g^−1^ DW (Figure 5A). The 1% sucrose concentration was most favorable for biosynthesis of the other major CQAs, i.e., 3,5-diCQA (4.21 mg g^−1^ DW), 4,5-diCQA (3.76 mg g^−1^ DW), 1,4,5-triCQA (3.67 mg g^−1^ DW), and 1,3-diCQA, occurring in the roots in an amount below 0.5 mg g^−1^ DW (Figure 4A and Figure 5A). Moreover, 1% sucrose was also the best for the production of flavonoids, with a total content of 4.4 mg g^−1^ DW (Figure 6A and Figure 7A). An increase in the sucrose concentration above 1% significantly reduced the flavonoid content.

The caffeoylquinic acid derivatives level lowered when 7% sucrose was used (Figure 3A, Figure 4A, Figure 5A and Figure 7A). It should be stressed that 3-CQA was only detected in the cultures treated with 7% sucrose (0.16 mg g^−1^ DW), while the sucrose-free medium was most efficient for producing 4-CQA (0.22 mg g^−1^ DW) (Figure 3A).

The level of chlorogenic acid (5-CQA; CHA) in *R. carthamoides* transformed roots increased with a sucrose concentration up to 3% and achieved a highest value of 4.91 mg g^−1^ DW (Figure 3A). Additionally, it was also shown that 3% sucrose stimulated the accumulation of 3,4-diCAQ and 1,5-diCQA in roots (Figure 4A). Higher or lower concentrations of sucrose significantly reduced the chlorogenic acid level in *R. carthamoides* transformed roots.

Our results showed that the optimal sucrose concentration was different for the biomass accumulation and biosynthesis of CQAs and flavonoids. Therefore, to obtain highly-productive cultures, with respect to the specialized metabolites, it is necessary to optimize the culture conditions regarding both the biomass and the specialized metabolite accumulation. Thus, in order to determine the best conditions for specialized metabolite productivity (expressed as yield in mg per liter of Woody Plant medium (WPM)), two parameters were assessed simultaneously, i.e., dry weight of *R. carthamoides* transformed roots and specialized metabolite level. Although the highest content of the main CQAs (sum of all identified CQAs) (30 mg g^−1^ DW) was recorded for roots growing in the presence of 1% sucrose (Figure 7A), the dry weight was four-times lower than for higher concentrations of the disaccharide (Figure 2B). The yield of these metabolites per liter of medium after five weeks was 243.5 mg L^−1^ (Figure 7B). The highest CQAs yield was noted for 3% sucrose, when the roots showed a high dry weight. One liter of medium could yield 511.1 mg of CQAs, i.e., 2–12-times more than in the other sucrose concentrations (Figure 7B).

Regarding the individual major CQAs, after five weeks, 144.33 mg L^−1^ of chlorogenic acid, 109.92 mg L^−1^ of the tentatively identified tri-CQA 1 derivative, and 85.24 mg L^−1^ of 4,5-diCQA were obtained from the roots treated with 3% of sucrose. Meanwhile, 100.75 mg L^−1^ of 3,5-diCQA was received from the roots cultured in the presence of 5% sucrose, and 29.8 mg L^−1^ of 1,4,5-triCQA from the roots treated with 1% of sucrose (Figure 3B, Figure 4B and Figure 5B). The greatest productivity of total identified flavonoids (38.9 mg L^−1^) was observed in the roots grown in WPM medium supplemented with 3% sucrose (Figure 7B).

The trend of changes in CQA and flavonoid content in *R. carthamoides* transformed roots treated with different sucrose concentrations was also presented graphically in a heat map, where the values of the individual metabolite contents were presented through the color intensity of the boxes (Figure 9A,B). A lighter color indicates a low metabolite content; while a darker color demonstrates higher metabolite levels.

In conclusion, 3% sucrose was found to give the best yield of CQAs (511 mg L^−1^) and flavonoids (39 mg L^−1^).

### 2.3. Growth Curve of Transformed Roots and Accumulation of Various CQAs

**Figure 10:**
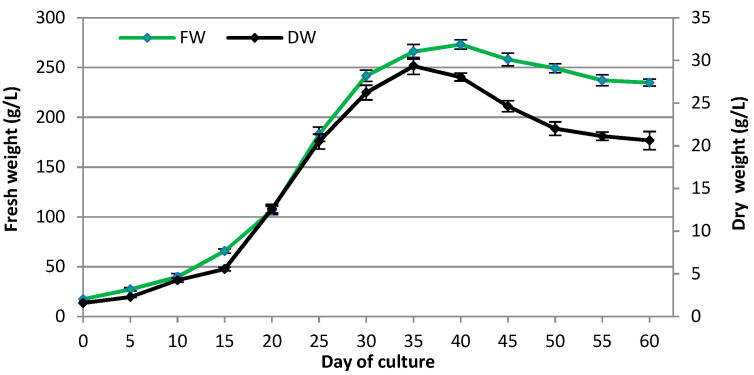
The changes of fresh and dry weight (g L^−1^) during the growth cycle of *R. carthamoides* transformed roots cultured for 60 days in 50 mL of liquid WPM medium supplemented with 3% sucrose in a 300 mL Erlenmeyer flask, in light conditions (16/8-h light/dark photoperiod) on a rotary shaker at 80 rpm. The results are means values ± SE of nine replicates for each time point of the growth curve.

**Figure 11:**
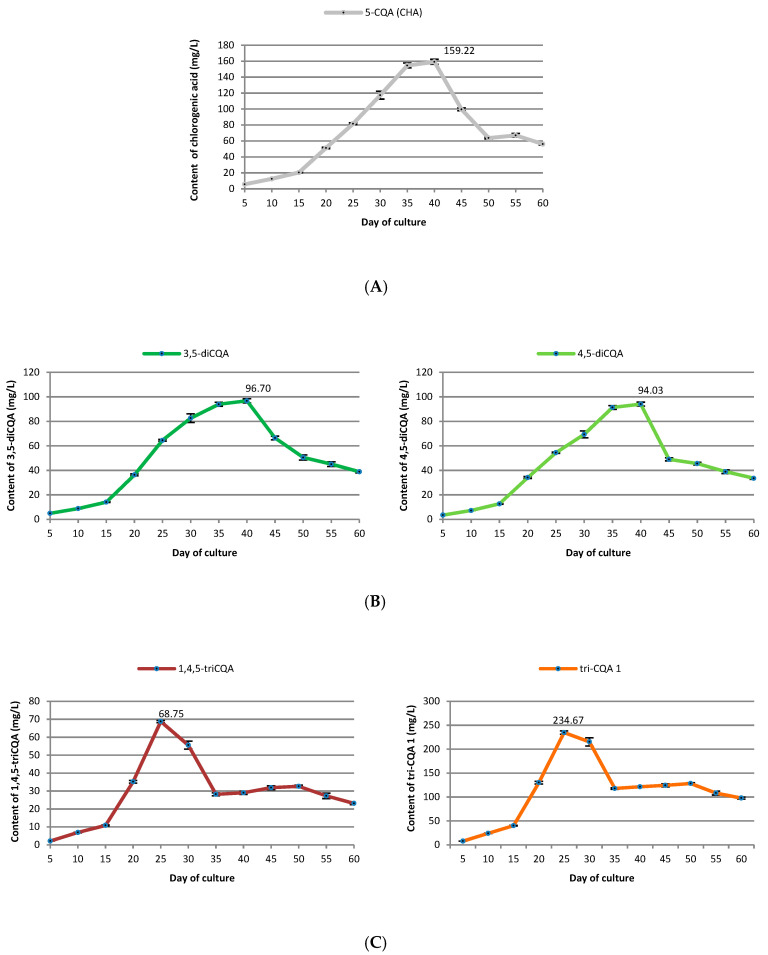
The changes in the content of caffeoylquinic acid derivatives (mg L^−1^) (**A**) 5-*O*-caffeoylquinic acid, (**B**) 3,5-di-*O*-caffeoylquinic acid and 4,5-di-*O*-caffeoylquinic acid, (**C**) 1,4,5-tri-*O*-caffeoylquinic acid and tentatively-identified tri-*O*-caffeoylquinic acid 1 derivative during the growth cycle of *R. carthamoides* transformed roots cultured for 60 days in 50 mL of liquid WPM medium supplemented with 3% sucrose in a 300 mL Erlenmeyer flask, in light conditions (16/8-h light/dark photoperiod) on a rotary shaker at 80 rpm. The results are means values ± SE of three replicates for each time point of the growth curve (*n* = 9). 5-CQA (CHA)-5-*O*-caffeoylquinic acid (chlorogenic acid); 3,5-diCQA-3,5-di-*O*-caffeoylquinic acid; 4,5-diCQA-4,5-di-*O*-caffeoylquinic acid; 1,4,5-triCQA-1,4,5-tri-*O*-caffeoylquinic acid; tri-CQA 1-tricaffeoylquinic acid derivative.

In order to estimate the changes in *R. carthamoides* transformed roots biomass and production of major CQAs (5-CQA; 3,5-diCQA; 4,5-diCQA; 1,4,5-triCQA, and a tentatively-identified tri-CQA 1) over time, the roots were cultured in liquid WPM medium supplemented with 3% sucrose (Figure 10 and Figure 11). The root biomass and CQA content were recorded at 5-day intervals over 60 days. The initial (lag) phase, when the biomass of the transformed roots increased slowly, occurred during the first five days of culture. After that, rapid increases in biomass were observed in an exponential growth phase, and after 20 culture days, the FW and DW had increased 6.1- and 8.2-fold over the initial biomass, respectively. A constant biomass increase was observed after the exponential growth phase, and the maximum FW and DW were achieved on day 40 (273.17 g L^−1^) and 35 (29.34 g L^−1^) of culture, respectively. Concerning the inoculum, the FW increased 14.7-fold and DW 16.1-fold (Figure 10). After this time, the biomass of the transformed roots gradually declined, and the stationary phase began. In the latter days of the growth cycle, the transformed roots became thicker and more fragile.

It was found that the maximum accumulation of individual CQAs varied according to the growth phase (Figure 11). The level of mono- and di-CQAs increased gradually during the growth curve of *R. carthamoides* transformed roots, and the profile accumulation of 5-CQA and the two di-CQAs was similar. The maximum values were achieved in day 40 (159.22 mg L^−1^ of 5-CQA, 96.7 mg L^−1^ of 3,5-diCQA, and 94.03 mg L^−1^ of 4,5-diCQA). The tri-CQAs demonstrated a sharp increase in content and the highest yields of tri-CQA 1 (234.67 mg L^−1^) and 1,4,5-triCQA (68.75 mg L^−1^) were on day 25 (Figure 11).

## 3. Discussion

In the present study, for the first time, the effect of different sucrose concentrations (0-7%) on the root growth and the biosynthesis of various CQAs and flavonoid monoglycosides in *R. carthamoides* transformed roots was evaluated. The changes in biomass and selected specialized metabolite accumulation in the transformed roots cultured in liquid WPM medium supplemented with an optimum sucrose concentration over a 60-day growth cycle were also investigated.

Many studies have indicated that the growth of transformed roots may be affected by the medium type (e.g., nutrients medium composition and salt concentration) and physical culture conditions (e.g., temperature, light intensity and wavelength, photoperiod or lack of light) [17,25]. A previous study on the influence of different liquid media (WPM, Schenk and Hildebrandt, Gamborg) with full- and half-strength macro- and microsalt concentrations and light conditions (growth in the dark or exposure to light; photoperiod 16h light/8h dark) on the growth of *R. carthamoides* transformed roots and CQA biosynthesis found that the medium type and strength, as well as the physical conditions, affected root growth [15]. Lower salt levels and dark conditions decreased both the fresh and dry weights of *R. carthamoides* transformed roots. The most favorable medium for biomass accumulation was WPM medium with the full macro- and microsalt concentration. The light conditions also affected the level of CQAs. It should be stressed that only light-grown hairy roots biosynthesized flavonoid glycosides, as quercetagetin, quercetin, luteolin, and patuletin hexosides [15].

The growth of transformed roots and the biosynthesis of the bioactive compounds can be determined using other factors, such as the type and the concentration of the carbon sucrose [23,26,27]. Sucrose, a disaccharide, is the most common source of carbon used in plant in vitro culture [28] because it is a cheap and readily available ingredient [29] and remains stable during autoclaving, without caramelization [28]. As plant in vitro cultures are heterotrophic, the medium must be supplemented with carbohydrates as a source of energy [27,28]. Moreover, sucrose is also responsible for maintaining the cell osmotic potential [29]. It influences the primary metabolic pathways, cell growth, differentiation, and development, and is involved in the regulation of many plant genes and their response to stress [30,31]; it also plays a role in plant culture growth and specialized metabolite production [25]. Sucrose non-fermentating-1-protein kinase-1 (SnRK1 protein kinase) is responsible for the production of sucrose and starch, and enzyme activity, and thus controlling the carbohydrate metabolism in plants and their development and growth [32]. Over-expression of SnRK1 in the leaves of transgenic tobacco plants elevated sucrose, glucose, and fructose levels [33]. Upregulation of glucose pyrophosphorylase, sucrose synthase, and sucrose phosphate synthase (sucrose metabolism genes) was also found to influence primary growth and biomass production in tobacco [34,35].

In the present study, it was observed that the sucrose concentration influenced the transformed root biomass of *R. carthamoides* and modified the phenolic compound metabolic pathway. However, the dynamics of both processes varied significantly.

### 3.1. Influence of Sucrose Concentration on Transformed Root Growth

The current study revealed that sucrose concentration affected both the morphology of *R. carthamoides* transformed roots and their fresh and dry weights. The thickest, longest, and most branched roots were those that grew in liquid WPM medium with full macro- and microsalt medium supplemented with a 3% sucrose concentration. They had the most compact structure. The transformed roots were green in color (Figure 1B). Lower and higher sucrose concentrations in WPM medium resulted in thinner, shorter, and less branched transformed roots. Similar results were observed by Petrova et al. [27] for *Arnica montana* transformed roots growing in a medium enriched with 3% sucrose; these were longer and more branched than those cultured in agar-solidified Murashige and Skoog medium containing 1% and 5–9% sucrose.

The fresh weight of *R. carthamoides* transformed roots increased with the sucrose concentration up to 3% and achieved optimal value of 265.87 g L^−1^. Previous studies of *Arnica montana* [27], *Plumbago europea* [36], and *Codonopsis pilosula* transformed roots [37] also found 3% sucrose to be the best concentration of carbon source for the fresh weight of roots. In the current study, it was found that higher sucrose concentrations (5% and 7%) significantly decreased the fresh weight of *R. carthamoides* transformed roots. Moreover, sucrose concentrations over 7% inhibited the biomass accumulation of roots and yielded 136.87 g L^−1^ FW and 23.54 g L^−1^ DW. This phenomenon may be related to the osmotic stress and/or toxicity of carbohydrates at higher concentrations [27,38]. The genes responsible for the induction of osmotic stress are upregulated by reactive oxygen species (ROS) including the transcription factor *DREB2A* (dehydration-responsive element-binding protein 2A) and a histidine kinase [39]. The osmotic stress may generate elevated ROS levels, which disrupt proper cell function, by damaging macromolecules such as membrane proteins, lipids, and DNA, thus influencing the morphological, physiological, and metabolic processes, and ultimately resulting in cell death [39,40]. Higher sucrose concentrations can enhance the viscosity of the liquid medium used in plant in vitro cultures [41] and thus can limit the availability of nutritional components of the medium needed for cell growth [29]. Sucrose can be rapidly hydrolyzed to glucose and fructose, resulting in an increase in medium osmolality [42]. Previous studies also confirmed that a higher initial concentration of sucrose in medium had no nutritional benefit; however, it caused osmotic stress and inhibited cell growth and biomass accumulation by transformed root cultures [23,37,43]. When sucrose was removed from the medium, the biomass of *R. carthamoides* transformed roots reached only 56.6 g L^−1^ FW. *R. carthamoides* transformed roots grew also slowly in the medium supplemented with sucrose at a concentration of 1%, most likely as a result of a lack of an energy source [44]. Low sugar concentrations or the lack of a carbon source in the plant culture medium also adversely affected biomass accumulation in other plant species transformed roots, such as *Centaurea calictrapa* [43], *Angelica gigas* [20], and *Picrorhiza kurroa* [23]. For example, 1% concentrations of different sources of the energy, such as sucrose, maltose, and glucose, had the weakest effect on the growth of *Arnica montana* transformed roots. After 40 days, the FW amounted to only 0.5 g, and this was seven-times less than after using sucrose at an optimal concentration (3%) [27].

In the current study, the dry weight of *R. carthamoides* transformed roots increased with sucrose concentration and achieved the highest value at a concentration of 7% (31.17 g L^−1^). In addition, 7% sucrose concentration also demonstrated the optimal dry biomass increase for *Panax quinquefolium* transformed roots cultured in a nutrient sprinkle bioreactor [21] and *Centaurea calictrapa* transformed roots growing in a shake flask [43]. It should be noted that 3% and 5% sucrose likewise increased the dry weight of *R. carthamoides* transformed roots in the present investigation, but with no significant difference with the 7% concentration. Although the fresh weight of *R. carthamoides* transformed roots was highest after treatment with 3% sucrose, the dry weight was comparable to that achieved for 5% and 7% sucrose. The high fresh weight increase of the roots may have been due to water accumulation in the cells; this may indicate a water balance in the cells or that the cells were deplasmolyzed. Additionally, it was noted that the dry matter percentage of *R. carthamoides* transformed roots peaked at 7% sucrose (Figure 2C), which indicated a lower water content in the cells and therefore a lower fresh weight of *R. carthamoides* transformed roots. Our results corroborate those reported for *Centaurea calictrapa* and *Panax ginseng* transformed roots [43,45].

### 3.2. Influence of Sucrose Concentration on CQA and Flavonoid Production

In the current investigation, HPLC-PDA analysis found that sucrose concentration (0–7%) had a significant influence on the level of CQAs and some flavonoid monoglycosides in the transformed roots of *R. carthamoides*. Sucrose can enhance the expression of key genes involved in the biosynthesis of phenolic compounds, such as phenylalanine ammonialyase (PAL), *p*-coumarate 3-hydroxylase (C3H), chalcone synthase (CHS), chalcone isomerase (CHI), and isoflavone synthase (IFS) [46,47]. CQAs are biosynthesized by the phenylpropanoid pathway: PAL produces cinnamic acid, which is converted to *p*-coumaroyl-CoA by cinnamate-4-hydroxylase (C4H) and 4-coumarate coenzyme A ligase (4CL). Subsequently, *p*-coumaroyl-CoA is converted to coumaroylquinate and then to CQAs by C3H. CQA can also be biosynthesized from caffeoyl-CoA and quinic acid by hydroxycinnamoyl CoA quinate hydroxycinnamoyl transferase (HQT) [48,49,50].

Only a few reports have been published on the effects of sucrose concentration on CQA biosynthesis in plant in vitro culture. Most of these are related to the accumulation of chlorogenic acid, which is one of the most abundant CQAs in plants [51,52,53,54]. In *R. carthamoides* transformed roots, the total CQAs content ranged from 2.30 mg g^−1^ DW to 30 mg g^−1^ DW, and the richest source of these compounds was the roots treated with 1% sucrose. The major components were tri-CQAs, which accounted for almost 60% of all identified CQAs, with mono-CQAs being only 12% (Figure 7A); this may be connected with the fact that mono-CQAs participate in di-CQA and tri-CQA biosynthesis [50]. The level of a tentatively-identified tri-CQA 1 derivative treated with 1% sucrose was 12.55 mg g^−1^ DW. Apart from the influence on the main CQAs, 1% sucrose was also the best for the accumulation of the other predominant CQAs, i.e., 3,5-diCQA, 4,5-diCQA, 1,4,5-triCQA, and 1,3-diCQA. On the other hand, the optimal sucrose concentration for accumulation of di-CQAs in *Ilex paraguariensis* callus cultures was 3% [54]. The study of Grunennvaldt et al. [54] also noted a decreased level of di-CQAs (3,4-, 4,5-, and 3,5-diCQAs) and mono-CQAs (3-, 4-, and 5-CQAs) at higher concentrations of carbohydrate (6% and 9%); these findings agree with those of the present study. The content of the specialized metabolites in *R. carthamoides* transformed roots fell dramatically (besides 3-CQA) with 7% sucrose; this might be related to osmotic or carbonyl stress, or carbohydrate toxicity.

The highest accumulation of chlorogenic acid in *R. carthamoides* transformed roots was noted for 3% sucrose. A similar observation was reported by Li et al. [53] in cell suspension cultures of *Lonicera macranthoids,* and by Grunennvaldt et al. [54] in *Ilex paraguariensis* callus cultures. Higher or lower concentrations of sucrose lowered the content of chlorogenic acid in *R. carthamoides* transformed roots in the present investigation and in *I. paraguariensis* callus cultures [54]. The opposite results were noted for *Echinacea angustifolia* and *Hypericum perforatum* adventitious roots, where 5% or 7–9% sucrose, respectively, resulted in enhanced 5-CQA levels [51,52].

In the present study, it was shown that 1% sucrose concentration was also optimal for the biosynthesis of flavonoids (especially for quercetagetin and quercetin hexosides) in *R. carthamoides* transformed roots. A similar observation was found in adventitious roots of *Morinda citrifolia* [55]. In other plant cultures, such as adventitious roots of *Echinacea angustifolia* [56], *Gynura procumbens* [57], or cell suspension cultures of *Artemisia absinthium* [58], higher sucrose concentrations (5% and/or 7%) were more beneficial for the biosynthesis of this class of compounds. However, Baque et al. [55] found that a 9% sucrose concentration decreased the flavonoid production in *Morinda citrifolia* adventitious roots, as did the absence of sucrose in one-fourth strength MS medium.

Our study showed that the optimal sucrose concentration for the biomass accumulation of *R. carthamoides* transformed roots was not the most efficient for CQA and flavonoid biosynthesis; this is consistent with the results of other authors [51,55]. For example, in *Hypericum perforatum* adventitious roots, the highest biomass was achieved at 3% sucrose, with an optimal chlorogenic acid production at 7–9% [51]. The results of our research show that growth of *R. carthamoides* transformed roots might be carried out in a two-phase culture, i.e., in a growth medium with a high sucrose concentration for biomass accumulation and in a production medium with low sugar concentration that stimulates the biosynthesis of the specialized metabolites.

### 3.3. Growth Curve of Transformed Roots and Accumulation of Some CQAs

The changes in the biomass of *R. carthamoides* transformed roots and the content of the main CQAs were also studied during the root growth cycle. The plant material was collected at 5-day intervals, over 60 days. Maximum FW and DW were achieved on day 40 (273.17 g L^−1^) and 35 (29.34 g L^−1^) of the culture, respectively. Comparing the transformed root growth of other plant species capable of CQA biosynthesis, it can be concluded that the roots of *Echinacea purpurea*, similarly to the *R. carthamoides* transformed roots in the current research, achieved the highest biomass increase on day 35–40, but this resulted in only about 12–13 g L^−1^ DW [59,60]. *Cichorium intybus* and *Lactuca virosa* transformed roots achieved an earlier (on day 20 or 30, respectively) higher dry weight than *E. purpurea* transformed roots, i.e., about 19-20 g L^−1^ [61,62], but this is a 1.5-times lower value than that found in the present study for *R. carthamoides* transformed roots.

It follows from the *R. carthamoides* transformed root growth curve that the roots can be an efficient source of biomass after just 40 days, when 273 g of plant material can be obtained (fresh weight) from one liter of medium. This value is more than 13-times higher than that obtained for the roots of one plant after three months of gardening in the soil (about 20 g FW) [63]. Additionally, the *R. carthamoides* transformed roots showed a stable biomass accumulation in our lab over two years in culture, which may have significance in the future for industrial production of raw materials from in vitro culture. The transformed roots were also cultured in strictly defined conditions, regardless of the season. They showed a rapid increase in biomass in a relatively short time. Moreover, the transformed roots were genetically stable, which is important for the homogeneity of the raw materials. As plants may demonstrate genetic variation and/or differences in biosynthesis depending on the climatic zone, time of cultivation, and plant development phase, *R. carthamoides* transformed roots may be an attractive alternative source of raw materials. Such conservation is also important as the species is threatened with extinction due to the mass acquisition of plant materials and is listed on the red list in its natural habitat [9]. For centuries, this species has been used in the traditional medicine of Siberia in cases of overstrain and common weakness after illness [6].

The highest level of chlorogenic acid, 4,5-diCQA, and 3,5-diCQA in *R. carthamoides* transformed roots was observed on day 40, when the roots had already reached their highest dry weight. On the other hand, the highest contents of tri-CQA 1 and 1,4,5-triCQA were on day 25 of culture, when the roots had not yet achieved the stationary phase.

The highest amount of chlorogenic acid (0.93 mg g^−1^) for *Echinacea purpurea* transformed roots was observed at day 40, when the roots reached their maximum dry biomass [59]. Abbasi et al. [60] observed that in another line of transformed roots of *E. purpurea*, the chlorogenic acid accumulation reached maximum value in the stationary phase, at day 40 (2.3 mg g^−1^). The highest level of chlorogenic acid in cell suspension cultures of *Lonicera macranthoids* was also found to be closely correlated with the maximum biomass growth of the cells, but in this case the maximum biomass was achieved on day 15 of the culture [53]. In the case of *Lactuca virosa* transformed roots, the maximum chlorogenic acid accumulation (1.5% DW) was noted earlier, in the logarithmic phase (at day 10) [61]. This value was similar to that for *R. carthamoides* transformed roots but was reached 30 days earlier. On the other hand, the chlorogenic acid level remained low throughout the growth cycle of *Gardenia jasminoides* cell suspension cultures (only 0.009 mg g^−1^ DW) [64].

The transformed roots of different plant species can produce CQAs other than chlorogenic acid, for example 3,5-diCQA, 4,5-diCQA or 3,4,5-triCQA [61,62,65]. The highest 3,5-diCQA content (5.57% DW) was observed on day 20 in *Cichorium intybus* transformed roots, which coincided with the highest dry biomass [62]. In addition, 3,5-diCQA concentration of 300 mg L^−1^ were observed for other line of *C. intybus* transformed roots after 6–12 days of culture [65]; this value was three-times higher than that found in the *R. carthamoides* transformed roots in the present study. In *L. virosa* transformed roots [61] and *Gardenia jasminoides* cell suspension cultures [64], the maximum 3,5-diCQA content was achieved at the beginning of cell growth, i.e., in the logarithmic growth phase.

## 4. Materials and Methods

The study used *R. carthamoides* transformed roots obtained by transformation with the *R. rhizogenes* A4 strain [15].

### 4.1. Influence of Sucrose Concentration on the Growth and Time Course of Transformed Roots

*R. carthamoides* transformed roots were cultured in liquid Woody Plant medium (WPM) [66], with a full macro- and microsalt concentration (without growth regulators), which was selected as optimal for root growth in our previous study [15]. WPM medium was supplemented with different sucrose concentrations (0%, 1%, 3%, 5%, and 7%). The cultures were grown in light conditions (16/8-h light/dark photoperiod; PPFD of 40 μmol m^−2^ s^−1^ under a cool-white fluorescent lamp) on a rotary shaker at 80 rpm. The roots, about 1 g fresh weight, were cultured in 300 mL Erlenmeyer flasks containing 50 mL of medium for 35 days. After this time, the transformed roots were dried on filter paper, and then the fresh weight (FW) and the dry weight (DW) (after lyophilization with using freeze dryer Alpha 1-2 LDPlus; M. Christ Gefriertrocknungsanlagen, Germany) were calculated and expressed in g L^−1^. The experiment was repeated in triplicate with three flasks of transformed roots for each treatment.

In order to determine the time course of changes in the growth of the transformed roots, the roots (0.867 ± 0.051 g FW) were cultured in 300 mL Erlenmeyer flasks containing 50 mL of liquid WPM medium with full macro- and microsalt concentration supplemented with 3% sucrose. The roots were harvested every five days during a 60-day growth cycle, and the FW and DW were calculated (g L^−1^). At each time point, three Erlenmeyer flasks were used. The experiment was repeated three times.

### 4.2. Effect of Sucrose Concentration on Metabolite Production

The contents of CQAs and flavonoid glycosides were determined in 80% (*v*/*v*) aqueous methanol extracts of transformed roots cultured in liquid WPM medium, with full macro- and microsalt concentration treated with different sucrose concentrations (0–7%) after 35 days in culture. Additionally, the specialized metabolite levels in the extracts were estimated during a 60-day growth cycle with five-day intervals for roots growing in analogous conditions with the optimized sucrose concentration (3%). The phytochemical analysis used material harvested from three flasks. The results were expressed in mg g^−1^ DW and mg L^−1^ of WPM medium.

The plant material extraction and phytochemical analysis were performed according to Skała et al. [15]. Briefly, the lyophilized transformed roots (500 mg) were ground and extracted three times, i.e., once with 25 mL and then twice with 10 mL of 80% (*v*/*v*) aqueous methanol, at 35 °C for 15 min, using an ultrasonic bath (InterSonic IS-20, Poland). Identification of compounds was performed using UPLC-PDA-ESI-MS^3^ and by comparison of their UV-Vis spectra and the retention times, according to our earlier study [15]. The quantitative studies of individual phenolic compounds were performed with HPLC-PDA analyses, using the same procedure as described previously [15]. The compounds were quantified as equivalents of chlorogenic acid (chlorogenic acid isomers), cynarin (dicaffeoylquinic acid isomers, tricaffeoylquinic acid and its derivative), and isoquercitin (flavonoid monoglycosides), and expressed in mg g^−1^ dry weight (DW) and mg L^−1^ of WPM medium.

### 4.3. Statistical Analysis

The results are calculated as means ± SE. The normality of the data was determined using a Shapiro–Wilk test. One-way analysis of variance (ANOVA) with Tukey’s post hoc test were used to identify significant differences (*p* < 0.05) between the samples (Statistica 13.1 software, StatSoft, Krakow, Poland). A heat map visualization was created on the basis of metabolite content data using Microsoft Office Excel.

## 5. Conclusions

The present study demonstrated, for the first time, that the sucrose concentration influenced both the growth of the roots and production of some specialized metabolites in *R. carthamoides* transformed roots. The results revealed that the optimal sucrose level was different for the biomass accumulation and the biosynthesis of specialized metabolites. Regarding the biomass of the roots and accumulation of CQAs, a 3% sucrose concentration appears to be optimal for phenolic acid productivity; about 510 mg L^−1^ of CQAs and 40 mg L^−1^ of flavonoids can be obtained after five weeks. Moreover, the growth curve of the roots indicated that the highest levels of mono- and di-CQAs were accumulated on day 40 of culture, while the highest amount of tri-CQAs occurred on day 25.

*R. carthamoides* transformed roots may be an efficient source of CQA derivatives and may serve as a good source of specialized metabolites at a commercial scale, especially if the roots are cultured in bioreactors in a liquid medium. This production method is advantageous considering that *R. carthamoides* is an endangered species.

## Data Availability

Data are contained within the article.
